# Maternal Platelets—Friend or Foe of the Human Placenta?

**DOI:** 10.3390/ijms20225639

**Published:** 2019-11-11

**Authors:** Gerit Moser, Jacqueline Guettler, Désirée Forstner, Martin Gauster

**Affiliations:** Division of Cell Biology, Histology and Embryology, Gottfried Schatz Research Centre for Cell Signaling, Metabolism and Ageing, Medical University of Graz, 8010 Graz, Austria; g.moser@medunigraz.at (G.M.); jacqueline.serbin@medunigraz.at (J.G.); desiree.forstner@medunigraz.at (D.F.)

**Keywords:** platelets, placenta, pregnancy, preeclampsia

## Abstract

Human pregnancy relies on hemochorial placentation, including implantation of the blastocyst and deep invasion of fetal trophoblast cells into maternal uterine blood vessels, enabling direct contact of maternal blood with placental villi. Hemochorial placentation requires fast and reliable hemostasis to guarantee survival of the mother, but also for the neonates. During human pregnancy, maternal platelet count decreases gradually from first, to second, and third trimester. In addition to hemodilution, accelerated platelet sequestration and consumption in the placental circulation may contribute to a decline of platelet count throughout gestation. Local stasis, turbulences, or damage of the syncytiotrophoblast layer can activate maternal platelets within the placental intervillous space and result in formation of fibrin-type fibrinoid. Perivillous fibrinoid is a regular constituent of the normal placenta which is considered to be an important regulator of intervillous hemodynamics, as well as having a role in shaping the developing villous trees. However, exaggerated activation of platelets at the maternal-fetal interface can provoke inflammasome activation in the placental trophoblast, and enhance formation of circulating platelet-monocyte aggregates, resulting in sterile inflammation of the placenta and a systemic inflammatory response in the mother. Hence, the degree of activation determines whether maternal platelets are a friend or foe of the human placenta. Exaggerated activation of maternal platelets can either directly cause or propagate the disease process in placenta-associated pregnancy pathologies, such as preeclampsia.

## 1. Hemochorial Placentation

**Figure 1:**
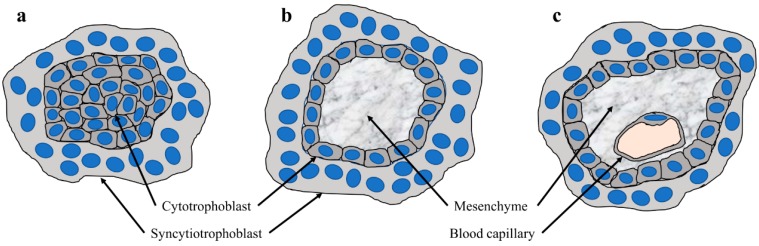
Development of placental villi (**a**) Section through a primary villus which shows a core of cytotrophoblasts covered by the syncytiotrophoblast; (**b**) section through a secondary villus, which shows a core of mesenchyme covered by a two-layered trophoblast epithelium, consisting of an inner, mononucleated cytotrophoblast layer, and the outer syncytiotrophoblast; and (**c**) section through a tertiary villus which shows blood capillaries in the mesenchyme.

At term, the human placenta is, by definition, “hemochorial” which means that maternal blood has direct contact with the trophoblast layer of the fetal placenta, however, before the hemochorial placenta is fully established, a number of tightly regulated developmental processes occur in the absence of maternal blood. One fundamental step is embryo implantation, which is initiated by the apposition of the blastocyst with its embryonic pole to the endometrial epithelium. As soon as the blastocyst establishes close and stable contact with the epithelium, the development of the placenta begins. Only the outer layer of the blastocyst, i.e., the trophoblast, is the precursor of the placenta, whereas, the embryoblast, umbilical cord, amnion, and embryo are derived from the inner cell mass [1]. During implantation (day six to seven post conception), the trophoblast starts to proliferate and fuse to a temporary invasive syncytiotrophoblast. Via the fusion of underlying cytotrophoblasts, the syncytiotrophoblast is continuously expanding around the embryo. This results in a spherical shaped placenta between day eight to 13 post conception (p.c.). Around day eight p.c., the first vacuoles form within the syncytiotrophoblast, which later fuse to bigger lacunae, separated by trabeculae. These lacunae are already filled with liquid (putative a mixture of glandular secretions, maternal blood, and blood plasma) and are the precursor of the intervillous space. The underlying cytotrophoblasts continuously proliferate and, at approximately day 14 p.c., finally migrate into the trabeculae. The trabeculae are filled with mononucleated cytotrophoblasts, covered with multinucleated syncytiotrophoblast, termed primary villi. Cytotrophoblasts can invade out of these primary villi, as extravillous trophoblasts (EVTs), into the maternal tissue and, from here on, the villous stage of placentation starts. Subsequently, mesenchymal cells follow the cytotrophoblasts within the primary villi and replace the cytotrophoblast core. There is always a layer of syncytiotrophoblast and cytotrophoblast, or a trophoblastic cell column between the mesenchymal cells and the maternal tissues, which are now termed secondary villi. As soon as the vasculogenesis within the mesenchyme starts (day 18 to 20 p.c.), the secondary villi turn into tertiary villi. Until the end of the first trimester of human gestation, nearly all villi turn into tertiary villi due to the distinct and branching vasculogenesis (Figure 1). The latter is mainly enabled by the intraplacental low oxygen concentration during the first trimester [2,3].

Intraplacental low oxygen concentration is crucial for proper placenta development and vasculogenesis and is the consequence of trophoblast plugs within the uterine spiral arteries, during early placental development. Trophoblast plugs result from EVT invasion in the spiral arteries, which leads to conversion of the vessels, including depletion of smooth muscle cells in their wall and loss of their elastic lamina [4,5,6]. This finally leads to a dilation of the arteries and conversion into flaccid conduits as a guarantee for a constant uteroplacental blood flow later in pregnancy.

In parallel, EVTs also invade the decidual stroma, up to the first third of the myometrium, uterine glands, veins, and lymphatics. The invasion of the decidual stroma (interstitial EVTs) serves to attach the placenta to the uterus and interacts with the decidual stroma cells and the immune cells. Endoglandular EVT invasion enables histiotrophic nutrition to establish prior to the establishment of the uteroplacental blood flow, by connecting the uterine glands to the intervillous space so that the glandular secretion products can reach the early conceptus [7,8]. Endoglandular trophoblasts replace the glandular epithelium, and thereby open the uterine glands to the intervillous space. Most likely, the invasion of endoglandular trophoblast occurs at the edges of the developing placenta, and the rapid lateral extension of the developing placenta [9] leads to a continuous accession of new glands to the placenta [10,11]. At the time of implantation, there is already first contact between trophoblast and uterine glands [8,10]. In the past, discussions of EVT invasion in veins has been controversially, but it is now well described and may serve to drain debris and waste from the placenta [12,13,14]. It is important to note that the EVTs are invasive, but not proliferative anymore, and that they have high levels of expression of the major histocompatibility complex, class I, G (HLA-G) and no longer express cell cycle genes. Although there are different populations of EVTs, there is currently no final proof that different routes of EVT invasion comprise different genetic signatures of EVTs [15,16,17].

**Figure 2:**
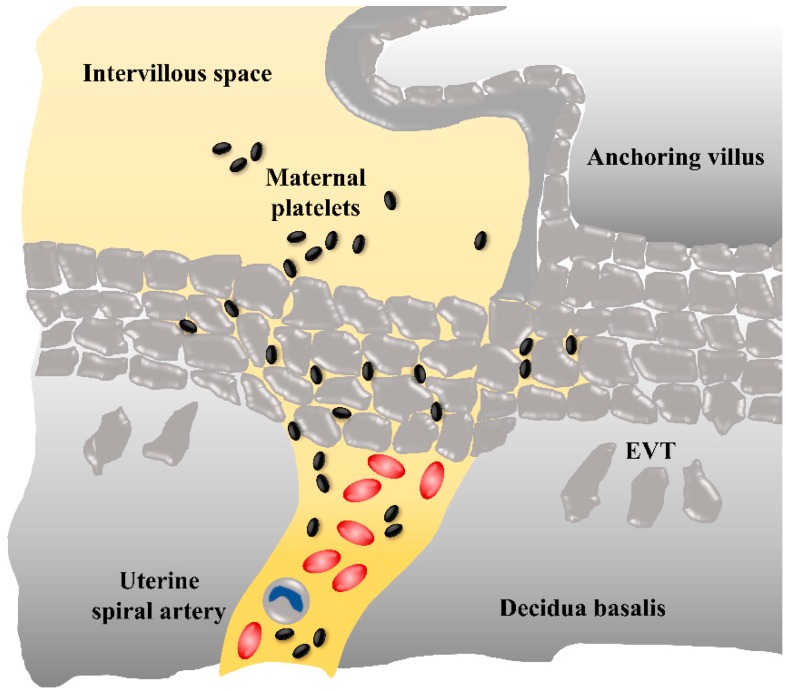
Potential route of maternal platelets into the early intervillous space. During early placenta development, maternal blood flow in uterine spiral arteries is obstructed by plugs of invaded extravillous trophoblasts (EVTs), however, maternal blood plasma and platelets can pass through narrow intertrophoblastic gaps, whereas erythrocytes and leukocytes are refrained from the intervillous space.

Nevertheless, during early pregnancy, plugs of invaded trophoblasts within the lumen of spiral arteries hinder the maternal blood cells from reaching the intervillous space. Detailed descriptions of trophoblast plugs have shown that such a plug can be comprised of at least 700 single EVTs over a distance of 700 µm, leading to a visible stowing of erythrocytes [18]. On the one hand, a computational simulation model has confirmed that the physiological plug structures are dense enough to restrict the flow of oxygenated blood to the intervillous space in the first trimester [19]. On the other hand, it has been demonstrated that, during the middle of the first trimester, narrow channels begin to form within the trophoblast plugs [20]. An extended computational model of trophoblast plugs has been designed to analyze possible behaviors of trophoblast cells as simply as possible, while incorporating key postulated modulators of this behavior [21]. The model estimated varying porosity of the plug, with no apparent relationship between plug porosity and gestational age. One of the variable settings demonstrated that in the presence of a background flow (e.g., from the maternal side towards the plug) trophoblasts are pushed into small clusters, and therefore, in general, the plug appeared as a coherent mass, but within this mass, there were regions with high cell density and regions in which cells were sparse. These regions with a sparse cell density (“weak spots”) could be those channels in the plug structure. This concept has been supported by a comprehensive morphological survey of spiral arteries showing only loosely cohesive plugs at week six, with clear capillary-sized channels in the intervillous space by seven weeks of gestation [20]. Moreover, contrast-enhanced ultrasound examinations across the first trimester in human subjects have recently demonstrated filling of the intervillous space from six weeks of gestation onwards [20,22]. Due to their small size, with a largest diameter of only 2 to 3 µm, maternal platelets can be the first among the maternal blood cells which enter the intervillous space. We, and others, have observed maternal platelets in fragmentary trophoblast plugs of uterine blood vessels [23], which suggests that platelets can pass through the narrow intercellular gaps of such weak trophoblast spots and enter the intervillous space even before uteroplacental blood flow is completely established (Figure 2).

**Figure 3:**
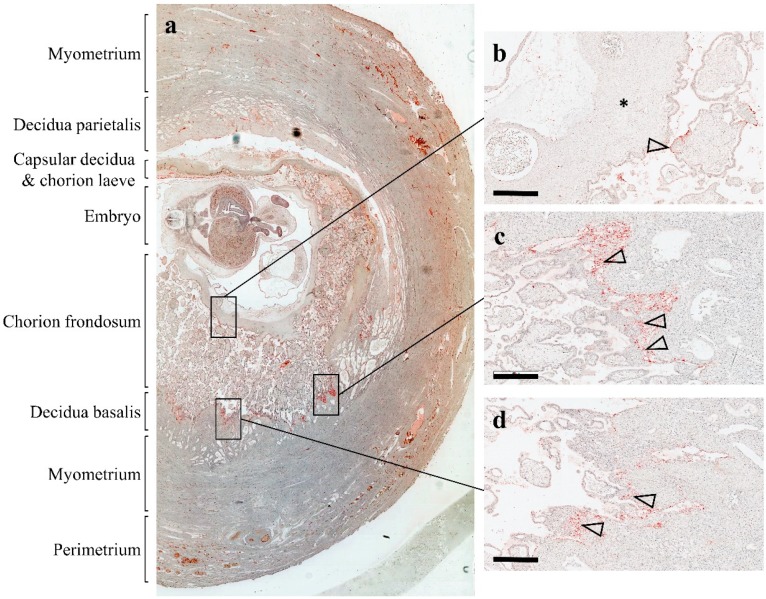
Localization of platelets in human first-trimester placenta in utero. An archival first-trimester human placenta in utero was obtained from hysterectomy and stained for platelet marker CD42b, as previously described by [24]. (**a**) The overview of the specimen shows the uterine wall composed of the perimetrium, myometrium, and endometrium, which after implantation is referred to as decidua basalis (invaded decidua) and decidua parietalis (noninvaded decidua). The chorion frondosum comprise developing placental villi, whereas the chorion laeve (smooth chorion) is the result of villous degeneration and obliteration of the intervillous space. (**b**) Maternal platelets can be detected on the syncytiotrophoblast layer of a villus (arrowhead) adjacent to the chorionic plate (asterisk). (**c** and **d**) An accumulation of platelets (arrowheads) can be detected in anchoring parts of trophoblast cell columns, which attach anchoring villi to the basal plate. Scale bars in **b** to **d** represent 400 µm.

In line with this assumption, we detected adherent platelets on the villous surface of first-trimester placenta tissue [24]. Our analysis of placental explant homogenates and correspondingly conditioned culture media revealed considerable levels of well-described platelet-derived factors, including chemokine (C-C motif) ligand 5 (CCL5) and chemokine (C-X-C motif) ligand 4 (CXCL4). Because CCL5 and CXCL4 are not synthesized in the villous trophoblast layer, adhering platelets seemed to be the only source for both chemokines detected in a conditioned culture media. Our observations suggest that platelets and their cargo represent an important regulator of early human placenta development. The localization of maternal platelets, in a unique archival specimen from a human first-trimester placenta in utero [10], shows maternal platelets not only on the surface of placental villi, but also in anchoring parts of trophoblast cell columns (Figure 3). It is not known, to date, whether platelet-derived factors affect the behavior of different trophoblast subpopulations, however, accumulation of platelets in intercellular gaps of distal parts of trophoblast cell columns raise the question as to whether they contribute in some way to trophoblast differentiation into the invasive phenotype. Likewise, adherence of maternal platelets on the villous surface and degranulation of cargo proteins could affect aspects of villous trophoblast physiology, including maternal-fetal transport and endocrine activity.

## 2. An Effective System for Hemostasis is Key for Hemochorial Placentation

The evolution of an invasive, hemochorial placentation solved at least two problems which include: (a) uterine inflammation caused by blastocyst attachment and subsequent penetration into the endometrial stroma, and (b) hemostasis. As mentioned above, hemochorial implantation includes invasion of EVTs into decidual blood vessels, with the important effect of maternal spiral artery remodeling to wide-bore, low resistance conduits. However, erosion of decidual blood vessels during implantation raises the question of how the bleeding is handled and limited to the area of placentation. Another challenge in hemochorial placentation arises at parturition, when the placenta is dissociated by uterine contractions, leaving a broad unprotected lesion in the uterine cavity. From an evolutionary point of view, fast and reliable hemostasis at the placental bed is not only essential for the survival of the mother, but also for the neonates. Since mammalian neonates rely on lactation for survival, maternal death, thus, also leads to neonatal demise. Considering this information, Martin and Wagner recently proposed the interesting concept that a preceding evolution of platelets was necessary for the origin of invasive, hemochorial placentation. Hence, deeply invasive placentation in eutherian mammals has only been possible in animals that could handle the challenging hemostatic consequences of hemochorial implantation [25]. Platelets have shown a quantitative hemostatic advance over functional equivalent cells in other species, including larger nucleated thrombocytes that occur in reptiles and birds [26], or thrombocyte-like cells, such as coagulocytes in insects [27]. Platelets are small, anucleate cell fragments, resulting from cytoplasmic constrictions of megakaryocytes in the bone marrow. Their small size results in a large increase in cellular surface area and speed of granule secretion. An additional evolutionary advantage over nucleate thrombocytes is that in response to bleeding, megakaryocytes can increase their DNA content rapidly, producing even more active platelets with increased receptor density, more organelles per unit cellular volume, and increased capacity to produce prothrombotic proteins and reduce bleeding time [25].

In contrast, the evolution of platelets as a consequence of the unique hemostatic requirements in placentation cannot be supported, because platelets and their megakaryocyte progenitors in the bone marrow occur in all mammals, including egg-laying monotremes, and marsupials, which show only short embryo attachment. Thus, it is apparent that neither live birth nor the presence of a placenta accounts for the evolution of platelets in mammals [28].

## 3. Maternal Platelets in Pregnancy

According to several large, population-based studies, the maternal platelet count decreases by approximately 10% at term in uncomplicated pregnancies [28], and increases postpartum. A systemic review of 46 studies suggests that the decrease in mean platelet count occurs gradually from first, to second, and third trimester [29], and may involve dilution of platelets by plasma volume expansion. In addition to hemodilution, multiple additional physiological changes during pregnancy, such as accelerated platelet sequestration and consumption in the placental circulation, can contribute to the lower platelet counts [30]. Blood flow through the placenta, due to some aspects, is similar to blood circulation through the spleen. In the spleen, some blood flows directly from the arterioles to the venules, without intervening capillaries, while other blood is shunted into the low-pressure pools in the sinusoids. Plasma preferentially flows directly to the venules, described as “plasma skimming”, while the blood cells are sequestered in the sinusoids [31]. Accordingly, platelet counts in splenic blood are approximately seven-fold greater than platelet counts in peripheral blood [32]. In the placenta, maternal blood is shunted into the low-pressure pools in the intervillous space and the highly branched architecture of villous trees can cause sequestration of maternal platelets, similar to the splenic sinusoids. Platelets sequestered within splenic sinusoids return to the circulation, whereas platelets sequestered within the placental intervillous space are activated by local stasis, turbulences, or damage of the syncytiotrophoblast, and thus contribute to the formation of fibrin-type fibrinoid (see below).

The changes in mean platelet count, in pregnant women at delivery, have been demonstrated by a shift to the left in the histogram of the platelet count distribution. In some cases, platelet counts fall below the lower limit [29,33], which is considered as incidental thrombocytopenia, referred to by some authors as gestational thrombocytopenia. Women with incidental thrombocytopenia do not bear an increased risk for a poor pregnancy outcome or delivery of a thrombocytopenic offspring [28,34]. Thus, examination of an otherwise healthy pregnant women with mild thrombocytopenia diagnosed after the mid second trimester can be limited to careful examination of the presence of hypertension or proteinuria. Incidental thrombocytopenia usually remits within several days, up to two months after delivery [35].

## 4. Platelets and Their Role in Placenta Development and Function

During human embryo implantation, tissue factor (TF) and plasminogen activator inhibitor-1 (PAI-1) are ascribed key roles in creating a “hemostatic envelope” around the invading blastocyst, and therefore preventing local decidual hemorrhage [36,37]. At this very early stage of embryo implantation, the syncytiotrophoblast is equipped with an enzymatic endowment that enables crossing of the endometrial epithelium and penetration of the underlying stroma. There, the invading trophoblast breaches decidual capillaries embedded in decidual stroma that express high levels of TF [37]. TF acts as a receptor for factor VIIa, and once TF/VIIa complexes are formed, the extrinsic pathway of the coagulation cascade is initiated, ultimately leading to the generation of thrombin. The thrombin cleaves fibrinogen to soluble fibrin, which is cross-linked by the factor XIIIa, and it activates platelets through protease activated receptors (PARs) to form a fibrin clot. The fact that there is no human TF deficiency genetic disorder underlines the importance of TF to human survival and probably during human pregnancy. In mice, TF knockout fetuses die in utero, whereas the incorporation of a human minigene, expressing TF at only 1% of the wild-type level, rescues these TF knockout mice. However, low-TF expressing female mice mated with low-TF male mice show a considerable incidence of midgestational hemorrhage, and placentas of low-TF expressing embryos are abnormal and show numerous maternal blood pools in the labyrinth, suggesting an important role of TF in placenta maintenance [38]. In addition to TF, dysregulation of thrombomodulin (TM) and endothelial protein C receptor (EPCR) pathway in murine placenta provokes placental failure and embryonic loss during mid gestation [39]. Deregulation of coagulation proteases and subsequent activation of platelets through the protease-activated thrombin receptor PAR-4 is mechanistically associated with the reproductive function of TM, because maternal platelet deficiency or maternal PAR-4 deficiency partially abrogates the lethal phenotype of TM-deficient embryos [40]. These observations in mice suggest that exaggerated activation of coagulation, leading to PAR-4 dependent platelet activation is causative related to embryonic demise [39]. Interestingly, the early steps of placenta development seem to be disturbed by activated platelets, since inhibition of platelet activation only before establishment the TM-dependent phenotype, but not when the phenotype becomes detectable, rescues the affected embryos.

Platelets and the maternal coagulation system contribute to the generation of fibrin-type fibrinoid, mainly consisting of fibrin, which are different from matrix-type fibrinoid that are generated by secreted extracellular matrix proteins from extravillous trophoblasts [41]. Fibrin-type fibrinoid can be detected in areas where the intervillous space is not lined by the syncytiotrophoblast or the maternal endothelium. Moreover, so-called perivillous fibrin-type fibrinoid is located at the surface of placental villi, where fibrinoid focally replaced the villous syncytiotrophoblast. There are two accepted theories of how fibrin-type fibrinoid is generated in human placenta. The first includes initial degeneration of the syncytiotrophoblast, exposing extracellular matrix components of the basal membrane, which leads to activation of maternal platelets and local coagulation of the maternal blood. According to this theory, fibrin-type fibrinoid acts as a substitute for the discontinuous syncytiotrophoblast barrier. The second theory suggests that turbulence or stasis of maternal blood in the intervillous space induces perivillous aggregation of platelets and the degeneration of syncytiotrophoblast is only a secondary phenomenon. Meanwhile, there is convincing evidence that both mechanisms are valid, and it is now well-accepted that fibrin-type fibrinoid is a regular constituent of the normal placenta. Not only deposition, but also continuous clearance of fibrin-type fibrinoid are normal events in human placenta throughout pregnancy [42], and the turnover of fibrin-type fibrinoid seems to be an important regulator of intervillous hemodynamics, and in shaping the intervillous space and the villous trees [41]. Accordingly, fibrin-type fibrinoid deposition, in particular on the surface of stem villi, contributes to the mechanical stability of the placenta. During pregnancy, the syncytiotrophoblast of stem villi is almost completely replaced by fibrin-type fibrinoid and the degree of fibrin deposition positively correlates with the thickness of stem villi [43]. Moreover, maternal blood clotting and fibrin deposition at intervillous areas showing stasis or turbulent blood flow, could contribute to adapting the shape of villous trees, as well as the intervillous space, to intervillous blood circulation. Hence, newly formed villi either influence the intervillous circulation in a positive manner, supporting their own persistence, or cause local stasis or turbulences, resulting in blood clotting and subsequent degeneration of these newly formed sprouts [41]. On the basis of this concept, Kaufman et al. suggested that the deposition of fibrin-type fibrinoid is a tool to revise unfavorable villous branching [41].

In addition to shaping the microanatomy of placental villi and the intervillous space, perivillous fibrinoid is involved in maternal-fetal transfer. If the syncytiotrophoblast is degenerated or injured by mechanical forces, exposure of basal lamina molecules activate maternal platelets, leading to quick deposition of fibrin-type fibrinoid at the site of damage. In total these areas account for approximately 7% of the villous surface at term [44], and have been suggested as para-trophoblastic routes for a transfer of macromolecules bypassing the syncytiotrophoblast. Moreover, such areas can serve as sites for a transplacental passage of a number of cells from maternal to fetal blood circulation, and vice versa, giving rise to the phenomenon of fetal and maternal microchimerism, respectively [45,46,47].

While fibrin deposition is a normal process in human placentation, the direct contribution of platelets to this process remains speculative. Observations in platelet-deficient mouse models suggest that the essential role of fibrin in the placenta does not depend completely on its interaction with platelets and that platelet dependent hemostasis is not strictly required for successful reproduction [48]. This view is supported by the absence of placental abnormalities in a variety of other established mouse mutants with profound platelet defects, including the loss of PAR-3 and PAR-4 mediated platelet activation [49,50]. These studies with mutant mice are consistent with the clinical reports in humans, since pregnant women with severe platelet defects, such as Glanzman thrombasthenia [51], Bernhard–Soulier syndrome [52,53], or a congenital absence of platelets [54] largely undergo successful pregnancies until term. Although the available data suggest that platelets are rather dispensable for successful reproduction, this should not be misinterpreted as an indication that maternal platelets have no function at all during pregnancy [55]. A growing body of evidence suggests a non-hemostatic platelet dependent function, during placentation [56]. This type of non-hemostatic function could relate to promoting trophoblast invasion. Immunostaining of early human placental tissues has shown platelets within remodeled maternal spiral arteries, where they attached to the surface of endovascular trophoblasts or to vessel walls that were infiltrated by perivascular trophoblasts [23]. The same study showed that CD41^+^ platelets adhere to CD146^+^ EVTs and that most of the platelets expressed P-selectin on the cell surface, indicating that they had been activated. Moreover, morphological observations showed a shift from spindled-shape to round integrin α1 expressing trophoblasts, when cocultured with platelets for a longer period, suggesting that platelet-derived soluble factors induced EVT differentiation toward the endovascular phenotype [23]. In this context, it has been suggested that EVT differentiation is mediated by activation of the chemokine receptor CCR1 in response to granule-stored CCR1 ligands such as RANTES (also referred to as CC-chemokine ligand 5, CCL5), MIP-1α (alias CCL3), and MCP-3 (alias CCL5). In addition, other platelet-derived factors such as epidermal growth factor (EGF), vascular endothelial growth factor (VEGF), and platelet-derived growth factor (PDGF) are also released upon platelet activation and seem to enhance trophoblast invasion [57,58]. According to their proposed concept, Sato et al. critically noted that activation of platelets in the spiral arteries requires the pre-existence of endovascular trophoblast aggregates, and thus activation of platelets is not the primary trigger of extravillous trophoblast invasion into the spiral arteries [59].

## 5. Platelets in Placenta-Associated Pregnancy Pathologies

In recent years, platelets have been linked with sterile inflammation, which is considered to be an inflammation in the absence of any infectious microorganisms. Accordingly, maternal platelets are activated by procoagulant extracellular vesicles, leading to adenosine triphosphate (ATP) release from platelets and inflammasome activation within trophoblasts through purinergic signaling [60]. Consequently, it has been suggested that placental sterile inflammation further amplifies into systemic effects, including renal and endothelial dysfunction, causing gestational vascular diseases, such as preeclampsia (PE) and HELLP syndrome (hemolysis, elevated liver enzymes, and low platelet count) [55,60]. PE is a multisystemic disorder, which complicates 3% to 5% of all pregnancies, characterized by de novo hypertension and proteinuria occurring after 20 weeks of gestation in a previously normotensive woman [61,62]. While the precise etiology of PE is still not clear, there seems to be common agreement that it is a placenta driven disorder, frequently associated with aberrant placental perfusion and the subsequent release of factors that trigger the maternal systemic inflammation, vascular endothelial dysfunction, and platelet activation. Women with PE may develop the HELLP syndrome, which occurs in 0.5% to 0.9% of all pregnancies and in 10% to 20% of women with severe PE [63]. The association of excessive platelet activation and consumption in PE has been suggested for many years [36,64,65]. Interestingly, platelet activation can occur several weeks prior to the clinical onset of PE [66], and increased mean platelet volume (MPV) in the late first trimester of gestation has been suggested to predict intrauterine growth restriction (IUGR) and PE [67]. In line with these studies, a recent meta-analysis, which included 69 articles, showed that platelet activation marker P-selectin and MPV, are significantly increased in women with PE as compared with controls [68]. According to longitudinal studies of changes in platelet size during human gestation, the increase in MPV in pregnant women with preeclampsia occurs gradually and can be detected approximately four to five weeks prior to the development of the disease [69,70]. The MPV increases when platelets become activated and large platelets are more adhesive and more likely to aggregate than small ones [71]. Platelets that are larger than that of normal platelets are more biologically active than platelets produced under steady-state conditions, and therefore may represent a way to provide a maximally effective response to a pathophysiological situation [28]. Moreover, features and reactivity of maternal platelets change in gestational vascular diseases, showing increased membrane fluidity and cholesterol concentration, and an increased ratio between unsaturated and saturated fatty acids, as well as an increased tendency to secrete ATP [72,73,74].

**Figure 4:**
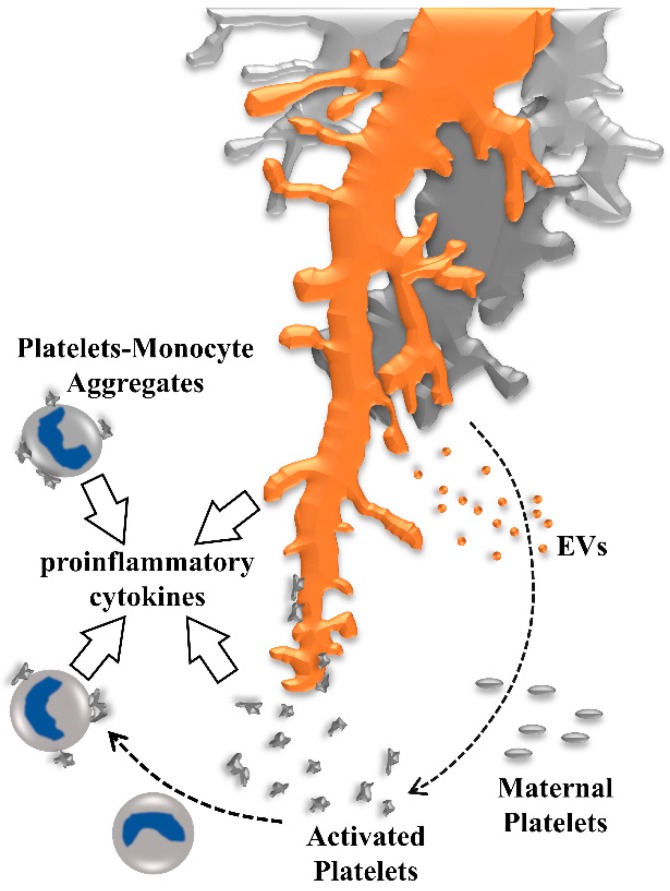
Activation of maternal platelets contributes to a proinflammatory environment in human placenta. Maternal platelets are activated by their passage through the intervillous space by local stasis, turbulences, or damage of the syncytiotrophoblast, moreover, platelets are activated by syncytiotrophoblast-derived extracellular vesicles (EVs) which are shed from the syncytiotrophoblast layer in response to oxidative and inflammatory stress. Activated platelets release ATP and provoke inflammasome activation in placental villi, leading to the release of proinflammatory cytokines into the intervillous space. Adhesion of activated platelets to the villous surface contributes to deposition of perivillous fibrin-type fibrinoids, which has been suggested as an important regulator of intervillous hemodynamics, and in shaping the microanatomy of villous trees. As soon as utero-placental blood flow is fully established, formation of platelet-monocyte aggregates induces monocytes to release proinflammatory cytokines and the antiangiogenic soluble fms-like tyrosine kinase (sFlt-1).

In pregnancies that are complicated by gestational vascular diseases, the oxidative and inflammatory syncytiotrophoblast layer of the placenta can shed increased numbers of extracellular vesicles with altered phenotype and cargo into the maternal circulation [75], which in turn could activate maternal platelets passing through the intervillous space (Figure 4).

This assumption is based on experimental evidence showing that syncytiotrophoblast-derived extracellular vesicles cause platelet activation, which is increased when extracellular vesicles were isolated from PE pregnancies [76]. Furthermore, pretreatment of platelets with aspirin, at concentrations similar to that in patients on low-dose aspirin (50 to 150 mg/day), abolished platelet aggregation caused by syncytiotrophoblast-derived extracellular vesicles in vitro. Meta-analyses of randomized controlled studies revealed that low-dose aspirin treatment, when started ≤16 weeks, significantly reduced the risk for severe PE [77,78]. Thus, in vitro data suggest that some of the clinical benefits of early low-dose aspirin administration in pregnancies with increased risk of placenta-associated complications are mediated by blocking extracellular vesicles induced platelet aggregation in vivo. In addition to aspirin, other platelet inhibitors, such as thromboxane A2 (TXA2) synthase inhibitors, TXA2 receptor antagonists, and 5-hydroxytryptamine receptor type 2 blockers have been considered. Nevertheless, experience in pregnancies complicated by gestational vascular diseases is limited [55]. Clopidogrel has not been shown to have an adverse effect on the fetus, but adequate and well-controlled studies in pregnant women are lacking [79].

Intriguingly, activated platelets, showing P-selectin on their surface, have the potential to bind to neutrophils and monocytes via leucocyte P-selectin ligand-1 (PSGL-1). Among all leukocytes, monocytes show the highest affinity for P-selectin and formation of platelet-monocyte aggregates is involved in a number of pathophysiological processes. On the one hand, platelet binding to monocyte PSGL-1 increases monocyte release of proinflammatory cytokines, including tumor necrosis factor (TNF)-α, as well as interleukin (IL)-1β, IL-6, IL-12, and IL-8 [80]. On the other hand, TNF-α has been shown to shift the cytokine secretion profile of the human placenta towards increased levels of granulocyte-macrophage colony-stimulating factor (GM-CSF), CCL5, and IL10, which has been suggested as a protective mechanism by the placenta to sustain trophoblast function and dampen inflammatory processes in the intervillous space [81]. In addition to P-selectin and PSGL-1, the chemokine CX3CL1 (also referred to as fractalkine) and its receptor CX3CR1 are directly involved in the platelet–monocyte complex formation [82]. Importantly, the fractalkine/CX3CR1 axis has been shown to mediate monocyte to trophoblast adhesion [83,84], and fractalkine is increased in placentas from severe early-onset PE [85], possibly contributing to increased adhesion of platelet-monocyte aggregates. In nonpregnant women and healthy control pregnancies, only low percentages of monocytes associated with platelets are detected, while the proportion of circulating platelet-monocyte aggregates are significantly increased in women with preeclampsia [86]. In contrast to monocytes, the percentage of neutrophils associated with platelets is not significantly changed in PE. However, adhesion of platelets to monocytes induces expression and release of the antiangiogenic soluble fms-like tyrosine kinase (sFlt-1) [87], which is significantly increased in maternal serum prior to the onset of the symptoms of PE, and thus has been suggested as a predictive biomarker [88]. Platelet-monocyte aggregates isolated from PE produce higher levels of sFlt-1 as compared to healthy controls, which has been suggested to contribute to endothelial dysfunction and an inappropriate inflammatory response in PE [87].

## 6. Conclusions

Studies with mutant mouse models suggest that platelets are rather dispensable for appropriate placentation and successful reproduction, however, in this context, extrapolations from murine to human placentation should be drawn with some caution, since human placenta differs from mouse placenta to some extent in terms of its morphogenesis and endocrine functions [89]. Thus, observations in mouse should not be misinterpreted insofar that platelets have no function at all during pregnancy. In normal human pregnancy, maternal platelets can contribute to perivillous fibrin deposition, and thus could, at least indirectly, contribute to the shaping of the microanatomy of placental villi and the intervillous space. However, under conditions of oxidative and inflammatory stress, maternal platelets can acquire a pathophysiological importance. Excess platelet activation, at the maternal-fetal interface, can provoke inflammasome activation in the placental trophoblast, and trigger enhanced formation of circulating platelet-monocyte aggregates, resulting in sterile inflammation of the placenta and a systemic inflammatory response in the mother. Hence, the answer to the question as to whether maternal platelets are a friend or foe to the human placenta depends on their degree of activation, which could either directly cause or propagate the disease process in placenta-associated pregnancy pathologies.

While the available data suggest that excess platelet activation induces inflammation in the human placenta, knowledge about its consequence on pivotal placenta functions, such as endocrine activity, metabolism, and transplacental transport is rather limited. Thus, future directions should focus on the point of time when platelets gain access to the intervillous space of the early first-trimester intervillous space. Studies on the interaction of maternal platelets with different trophoblast subtypes, i.e., extravillous trophoblast, villous cytotrophoblast, and syncytiotrophoblast, could reveal different effects of platelet-derived factors on individual subtypes. Moreover, in vitro studies with trophoblast-platelet cocultures should consider fluidic flow and different oxygen conditions in culture.

## Abbreviations

ATP	adenosine triphosphate
CCL3	chemokine (C-C motif) ligand 3 (CCL3)
CCL5	chemokine (C-C motif) ligand 5
CCR1	C-C chemokine receptor type 1
CD146	cluster of differentiation 146
CD41	cluster of differentiation 41, integrin alpha-IIb
CD42b	cluster of differentiation 42b, platelet glycoprotein Ib alpha chain
CX3CL1	chemokine (C-X3-C motif) ligand 1
CX3CR1	CX3C chemokine receptor 1
CXCL4	chemokine (C-X-C motif) ligand 4
EGF	epidermal growth factor
EPCR	endothelial protein C receptor
EV	extracellular vesicles
EVT	extravillous trophoblast
GM-CSF	granulocyte-macrophage colony-stimulating factor
HELLP	hemolysis, elevated liver enzymes, low platelet count
HLA-G	major histocompatibility complex, class I, G
IL-12	interleukin-12
IL-1β	interleukin-1β
IL-6	interleukin-6
IL-8	interleukin-8
IUGR	intrauterine growth restriction
MCP-3	monocyte-chemotactic protein 3
MIP-1α	macrophage inflammatory protein 1-alpha
MPV	mean platelet volume
p.c.	post conception
PAI-1	plasminogen activator inhibitor-1
PAR	protease activated receptors
PDGF	platelet-derived growth factor
PE	preeclampsia
PSGL-1	leucocyte P-selectin ligand-1
RANTES	regulated on activation, normal T cell expressed and secreted
sFlt-1	soluble fms-like tyrosine kinase
TF	tissue factor
TM	thrombomodulin
TNF-α	tumor necrosis factor-α
TXA2	thromboxane A2
VEGF	vascular endothelial growth factor
